# α-Conotoxins and α-Cobratoxin Promote, while Lipoxygenase and Cyclooxygenase Inhibitors Suppress the Proliferation of Glioma C6 Cells

**DOI:** 10.3390/md19020118

**Published:** 2021-02-21

**Authors:** Tatiana I. Terpinskaya, Alexey V. Osipov, Elena V. Kryukova, Denis S. Kudryavtsev, Nina V. Kopylova, Tatsiana L. Yanchanka, Alena F. Palukoshka, Elena A. Gondarenko, Maxim N. Zhmak, Victor I. Tsetlin, Yuri N. Utkin

**Affiliations:** 1 Institute of Physiology, National Academy of Sciences of Belarus, ul. Akademicheskaya, 28, 220072 Minsk, Belarus; terpinskayat@mail.ru (T.I.T.); tanyaya190@gmail.com (T.L.Y.); efpoluko@list.ru (A.F.P.); 2 Shemyakin-Ovchinnikov Institute of Bioorganic Chemistry, Russian Academy of Sciences, ul. Miklukho-Maklaya 16/10, 117997 Moscow, Russia; osipov@mx.ibch.ru (A.V.O.); evkr@mail.ru (E.V.K.); kudryavtsevden@gmail.com (D.S.K.); takhizis90@mail.ru (N.V.K.); gondarenkoea@gmail.com (E.A.G.); mzhmak@gmail.com (M.N.Z.); vits@ibch.ru (V.I.T.)

**Keywords:** α-cobratoxin, α-conotoxin, cyclooxygenase inhibitor, glioma C6, lipoxygenase inhibitor, proliferation, real-time polymerase chain reaction, viability

## Abstract

Among the brain tumors, glioma is the most common. In general, different biochemical mechanisms, involving nicotinic acetylcholine receptors (nAChRs) and the arachidonic acid cascade are involved in oncogenesis. Although the engagement of the latter in survival and proliferation of rat C6 glioma has been shown, there are practically no data about the presence and the role of nAChRs in C6 cells. In this work we studied the effects of nAChR antagonists, marine snail α-conotoxins and snake α-cobratoxin, on the survival and proliferation of C6 glioma cells. The effects of the lipoxygenase and cyclooxygenase inhibitors either alone or together with α-conotoxins and α-cobratoxin were studied in parallel. It was found that α-conotoxins and α-cobratoxin promoted the proliferation of C6 glioma cells, while nicotine had practically no effect at concentrations below 1 µL/mL. Nordihydroguaiaretic acid, a nonspecific lipoxygenase inhibitor, and baicalein, a 12-lipoxygenase inhibitor, exerted antiproliferative and cytotoxic effects on C6 cells. nAChR inhibitors weaken this effect after 24 h cultivation but produced no effects at longer times. Quantitative real-time polymerase chain reaction showed that mRNA for α4, α7, β2 and β4 subunits of nAChR were expressed in C6 glioma cells. This is the first indication for involvement of nAChRs in mechanisms of glioma cell proliferation.

## 1. Introduction

Tumor cells are characterized by the activation of mechanisms that enhance proliferation and resistance to apoptotic signals. In many types of cancer, such as lung [1,2], breast [3] and stomach [4,5,6] cancer, and in a number of others, nicotinic acetylcholine receptors (nAChRs) are involved in the mechanisms of cancer progression. nAChRs are ligand-activated ion channels that allow sodium, potassium and calcium ions to pass through ion pore [7]. Depending on the location and function, nAChRs are divided into two types—muscle and neuronal ones [8,9,10]. The muscle-type nAChRs are located at the postsynaptic membranes of nerve–muscle junction and take part in transmission of signals for the muscle contraction. The neuronal nAChRs are expressed in the central and peripheral nervous system, taking part in the transmission of fast nerve impulses. nAChRs are also expressed in many types of non-neuronal cells where they regulate many cellular functions [11,12]. The role of nAChRs in non-neuronal cells is a subject of extensive studies, but it is already clear that these receptors are involved in the regulation of proliferation and migration of cancer cells [13,14], promote tumor formation [15] and enhance the resistance of tumor cells to apoptosis [14,16,17]. In addition, a number of experimental studies have shown a slowdown in tumor growth in vivo under the action of nAChR blockers such as α-conotoxins [18,19,20], α-cobrotoxin [21] and alkylpyridinium polymer APS8 [22]. However, the cytotoxic properties of nicotine, agonist of the most of nAChRs, against both normal [23,24,25,26] and tumor [27] cells are known. These facts determine the persistent interest in the role of nAChR in tumor pathogenesis and antitumor therapy.

Another pathway involved in the tumor survival and growth includes the activation of enzymes in arachidonic acid cascade—cyclooxygenases (COXs) [28] and lipoxygenases (LOXs) [29]. COXs participate in the synthesis of prostanoids, which are autocrine and paracrine mediators involved in many physiological reactions [30]. They are high-affinity agonists of receptors, belonging to the family of G-protein-coupled receptors, which are involved in inflammation and carcinogenesis (e.g., [31]). There are known two main COXs isoforms—COX-1 and COX-2. The former, a constitutive enzyme, is permanently present in tissues and ensures normal physiological functions, while COX-2 is an inducible enzyme and its increased expression is observed in inflammatory diseases and tumor growth [32]. COX-3 was identified as well; it is produced by alternative splicing of the COX-1 gene. The expression of COX-3 increases with the development of tumors in certain organs and tissues, including the brain, ovaries, cervix, colon, and with leukemia [33,34].

LOXs are involved in the synthesis of hydroxyeicosatetraenoic acids and leukotrienes. Six LOX isoforms were identified in humans, and seven in mice [29]. Proinflammatory and protumor roles of the compounds formed with the participation of 5-LOX [35,36,37] and 12-LOX [35,36,38] are well established. COX [39,40,41,42] and LOX inhibitors [35,40,43,44] reduce tumor formation and slow the growth of tumors in the experiment and in some cases in the clinic [39,40,41].

Gliomas originating from various brain glia cells are very malignant and poorly treatable tumors [45], and the study of the mechanisms of their growth and the search for new methods for treatment is urgently required. There are three types of gliomas: astrocytomas, oligodendrogliomas and ependymomas. Astrocytomas emerging in brain from astrocytes is very difficult to treat because they spread throughout the normal brain tissue and, like other malignant tumors, proliferate rapidly. For effective treatment, the knowledge of the molecular mechanisms underlying proliferation is necessary. The C6 glioma cell line, a rat cell line of astrocytic origin, is a well characterized, safe and most-used glioma model, reflecting the main characteristics of the highly malignant and aggressive brain glioblastoma (grade IV astrocytoma) multiforme [46]. There is evidence that compared to nontumor samples in the samples of patients with glioblastomas, increased expression of mRNA for some subunits of nAChRs, in particular, α1, α9 and β1 subunits, has been detected. Furthermore, expression of mRNA encoding α7 and β2 subunits was upregulated in areas of active invasion (leading edge) compared to tumor mass (cellular tumor) [47]. In the 1321N1 and A172 astrocytoma cell lines, the presence of homomeric α7 nAChR and heteromeric αxβy nAChRs was found [48]. Expression of subunits of both homomeric (α7) and heteromeric (α4, β2 and β3) nAChRs on primary cultures of rat astrocytes was demonstrated [49]. However, the expression of nAChRs in C6 glioma is poorly characterized. Previously, it was shown only that C6 glioma cells express α7 nAChR [50,51]. This justifies our interest in the nAChR containing the aforementioned several subunits. To study possible involvement of these receptors in survival and proliferation of glioma C6 cells, we chose the animal toxins that specifically block distinct nAChR subtypes. We have previously shown that the combined use of α-conotoxins, nAChR blockers, and COX and LOX inhibitors promotes the death of Ehrlich carcinoma cells in vitro and slows tumor growth in vivo [20]. It should be noted that Ehrlich ascites carcinoma has a non-neuronal origin and is a spontaneous murine mammary adenocarcinoma. The aim of the present study is to investigate the role of nAChRs and COXs/LOXs in the regulation of the proliferative activity and survival of rat C6 glioma cells. In this work, we investigate the effects of the separate and combined application of the nAChR antagonists and the COX/LOX inhibitors on the C6 glioma cells. As a result, we discover for the first time the involvement of nAChRs in mechanisms of glioma cell proliferation.

## 2. Results

### 2.1. Effect of nAChR Blockade on the Viability and Proliferation of C6 Glioma Cells

α-Conotoxins and α-cobratoxin (CTX) were used as well-known nAChR inhibitors [52]. As mentioned in the Introduction, C6 glioma cells express α7 nAChR [50,51]. In addition, according to the data for astrocytomas and astrocytes, they may be expected to express neuronal heteromeric nAChRs. Basic on these considerations, we chose several α-conotoxins selective to distinct nAChR subtypes (Table 1) and CTX, selective inhibitor of neuronal homomeric nAChRs of α7 [53] and α9α10 [54] subtypes, for the study.

**Figure 1:**
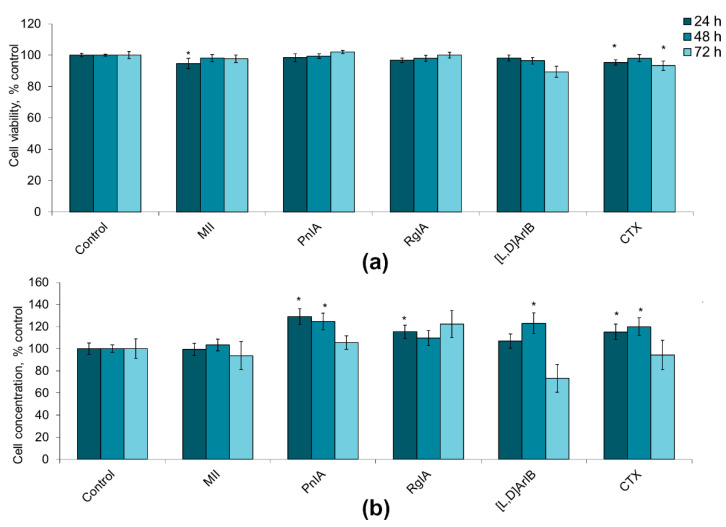
The effect of α-conotoxins MII; PnIA; RgIA; [V11L,V16D]ArIB ([L,D]ArIB) and α-cobratoxin (CTX) at concentration of 1 nM on the viability (**a**) and on the proliferative activity (**b**) of C6 glioma cells after 24 h (n = 20), 48 h (n = 20), and 72 h (n = 10–12) of incubation. The cell viability and concentration were determined by flow cytometry. * *p* < 0.05 according to Student’s *t*-test when compared to control.

To evaluate glioma cell viability and proliferation, flow cytometry was used. The cell viability was determined by staining with propidium iodide. Cell proliferative activity was established by calculating the cell concentration in the samples using reference fluorospheres. α-Conotoxins differing in the specificity towards distinct nAChR subtypes at a dose of 1 nM had little effect on the viability of C6 glioma cells, reducing this value by 1–11%, in most cases without statistical significance (Figure 1a). At the same time, their addition resulted in a certain increase in the proliferation of glioma cells, which was most clearly observed after 24–48 h. After treatment with α-conotoxin PnIA, the number of cells increased by 25–29%; α-conotoxin RgIA, by 10–15%; α-conotoxin [V11L,V16D]ArIB, by 7–23%; and CTX, by 15–20%, although not in all cases statistical significance was achieved (Figure 1b). After 72 h, the tendency to an increase in proliferation remained only in the case of α-conotoxin RgIA (the cell count increased by 22%). No effect of α-conotoxin MII on the proliferation of C6 glioma cells was found.

With an increase in the concentration of blockers to 100 nM, similar weakly expressed tendencies were found; viability decreased by 1–2% or did not change, proliferation in some series increased by 3–19% (Figure S1). The selective interactions of α-conotoxins with distinct nAChR subtypes are manifested at the concentrations well below 100 nM [52,53,54,55,56,57,58,59,61,62]. As the selectivity of toxins strongly decreases with an increase in concentration, the higher toxin concentrations were not studied.

**Figure 2:**
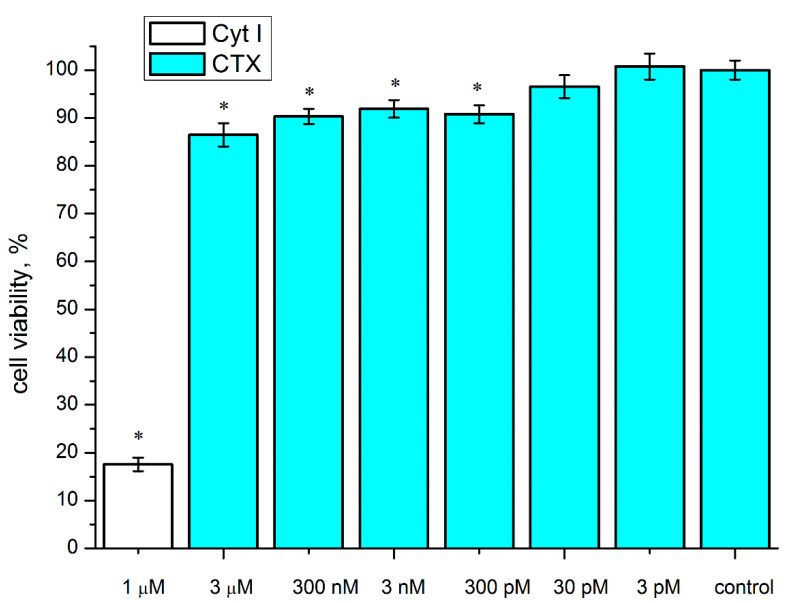
Effect of CTX on the survival of rat C6 glioma cells. The cells were incubated in the presence of various CTX concentrations (3 pM–3 μM) for 72 h. Cytotoxin I (Cyt I) from *Naja oxiana* venom was used for a positive control. The cell viability was determined by MTT test. Data are shown as % of control (untreated cells). The results are presented as the mean ± SEM in three (for concentrations of 3 nM and 300 pM—four) independent experiments with 12 replicates in each. * *p* < 0.05, Student’s two-sample *t*-test compared to control.

The most reliable, albeit small, decrease in the viability of the C6 cells was caused by the CTX. Therefore, using the colorimetric 3-(4,5-dimethylthiazol-2-yl)-2,5-diphenyltetrazolium bromide (MTT) test we analyzed the effect of different CTX concentrations on the survival of the C6 cells. Already at a CTX concentration of 300 pM, statistically significant reduction in the viability of these cells was found. However, a further increase in concentration up to 3 μM did not result in an increase in this effect (Figure 2).

**Figure 3:**
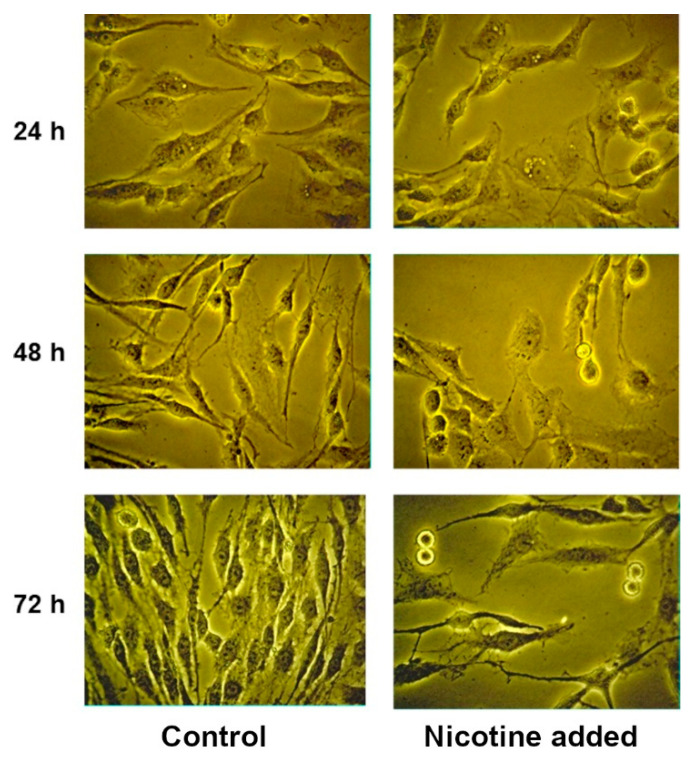
Glioma C6 cells treated with nicotine at a concentration of 1 μL/mL (6.1 mM).

We tested the effects of nicotine, which is an agonist for all nAChRs with the exception of α9 subtype for which it is antagonist. At concentrations of 0.001–0.1µL/mL (6.1 µM–0.61 mM), nicotine exerted no effect on the proliferative activity of glioma C6 cells and the loss of viability was 1–4% (Figure S2). Analysis by light microscopy showed that nicotine at concentrations of 1 µL/mL (6.1 mM) and higher induced morphological changes like cell rounding up and loss of processes followed by surface detachment (Figure 3). Despite these changes, we investigated the effect of nicotine at a concentration of 1 μL/mL (6.1 mM) on the proliferation and viability of C6 cells. At this nicotine concentration, inhibition of proliferation was observed, which after 72 h led to a decrease in the number of cells by more than two times; the viability was also reduced (Figure S2). However, it should be taken into account that the reason for such a strong decrease in the concentration of cells may be their detachment from the surface and, as a consequence, the cessation of division. We tested acetylcholine at concentrations ranging from 2 µM to 2 mM with incubation times of 24, 48 and 72 h. No effects of acetylcholine were observed.

### 2.2. Effect of COX Inhibitors and of Their Combinations with nAChR Blockers on the Viability and Proliferation of C6 Glioma Cells

**Figure 4:**
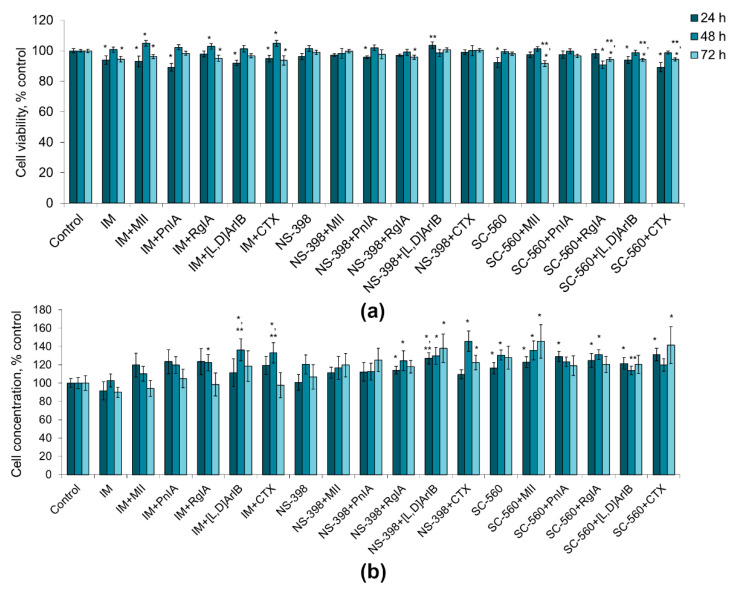
Effect of α-conotoxins MII; PnIA; RgIA; [V11L,V16D]ArIB ([L,D]ArIB) and α-cobratoxin (CTX) at concentration of 1 nM and COX inhibitors (IM, indomethacin, 10 µМ; NS-560, 5 µM; SC-398, 0.1 µM) applied separately or in the indicated combinations on viability (**a**) and on the proliferative activity (**b**) of C6 glioma cells after 24 h (n = 10), 48 h (n = 15) and 72 h (n = 10) of incubation. The cell viability and concentration were determined by flow cytometry. * *p* < 0.05 according to Student’s *t*-test when compared to control; ** *p* < 0.05 according to Student’s *t*-test when compared to the corresponding COX inhibitor.

Application of COX inhibitors alone and of their combinations with nAChR blockers did not produce a noticeable decrease in the viability of the C6 glioma cells (Figure 4a). The decrease in viability in most series was 1–11%, not always reaching statistical significance. Thus, COX inhibitors and their combinations with nAChR blockers have little or no effect on the viability of C6 glioma cells.

Indomethacin and NS-398 did not reveal statistically significant effects on the proliferative activity of the cells, although NS-398 showed a tendency to an increase in the number of cells by 20 and 7% after 48 h and 72 h of incubation, respectively (Figure 4b). SC-560 promoted proliferation, increasing the number of cells by 16–30%. When COX inhibitors were combined with nAChR blockers of various specificities, an increase or a tendency to an increase in the number of cells was observed (by 10–46%), which was the most pronounced when α7 nAChR blockers (CTX and [V11L,V16D]ArIB) were used (Figure 4b). Although in some cases statistically significant differences were observed in the action of COX inhibitors alone and COX inhibitors in the presence of nAChR blockers (e.g., IM versus IM+[V11L,V16D]ArIB or IM versus IM+CTX), in general there were no consistent differences between the two series.

### 2.3. Effect of LOX Inhibitors and of Their Combinations with nAChR Blockers on the Viability and Proliferation of C6 Glioma Cells

**Figure 5:**
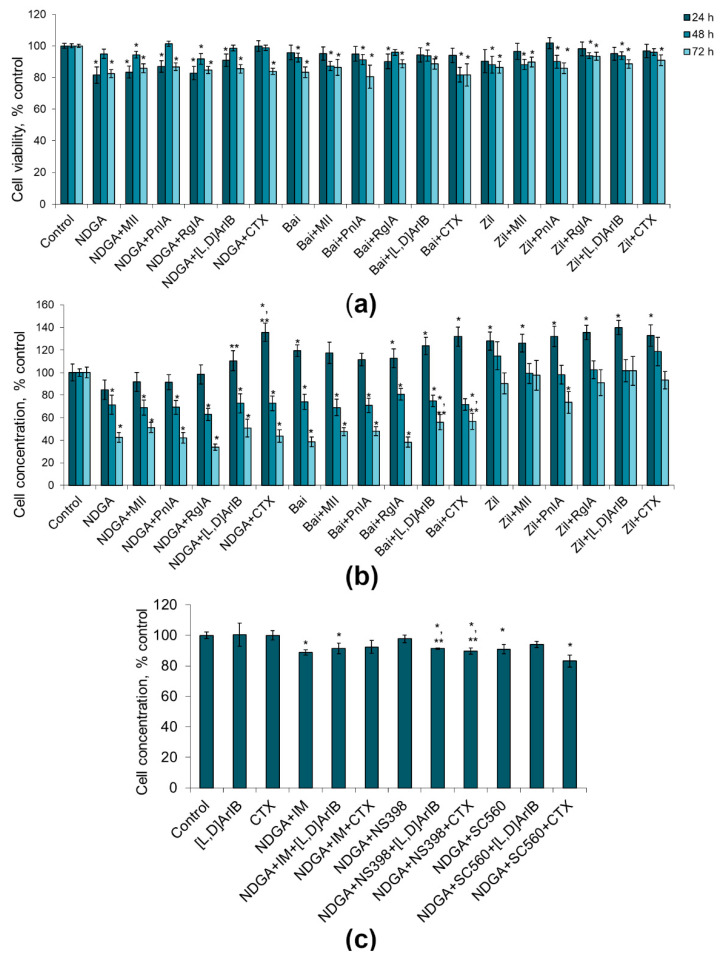
Effect of α-conotoxins MII; PnIA; RgIA; [V11L,V16D]ArIB ([L,D]ArIB) and α-cobratoxin (CTX) at concentration of 1 nM and LOX inhibitors (NDGA, nordihydroguaiaretic acid, 30 µM; Bai, baicalein, 50 µM; Zil, zileuton, 50 µM) on the viability (**a**) and on the proliferative activity (**b**) of glioma C6 cells after 24 h (n = 15), 48 h (n = 14) and 72 h (n = 15) of incubation. (**c**) Effect of the combined use of NDGA and COX inhibitors (IM, indomethacin, 10 µМ; NS-560, 5 µM; SC-398, 0.1 µM) with the α7 nAChR blockers on the proliferation of C6 glioma cells after 24 h of incubation (n = 5). The cell viability and concentration were determined by flow cytometry. * *p* < 0.05 according to Student’s *t*-test when compared to control; ** *p* < 0.05 according to Student’s *t*-test when compared with the corresponding LOX/COX inhibitor.

We observed that the effect of LOX inhibitors was time dependent. After 24 h of incubation, nordihydroguaiaretic acid (NDGA), a nonspecific lipoxygenase inhibitor, reduced the viability of C6 glioma cells by 18% (Figure 5a). When the combinations with α-conotoxins were used, there was a tendency to weakening the effect of NDGA (by 1–9%), while CTX canceled the effect of NDGA completely. Similar trends were observed in the effects on the cell proliferative activity: NDGA decreased the number of cells by 15%, whereas the nAChR blockers reduced this effect, and it was statistically significant only with α7 nAChR blockers, as compared to NDGA alone (Figure 5b). During 24 h cultivation, baicalein and zileuton and their combinations with nAChR blockers did not manifest cytotoxic and antiproliferative effects (Figure 5a,b). Moreover, the cells divided even more intensively than in the control experiments both with baicalein and zileuton, and under the combinations of NDGA with α7 nAChR blockers (Figure 5b).

After 48 h of cultivation, cell viability under the influence of LOX inhibitors decreased by 1–18%; the combined use of LOX inhibitors with nAChR blockers did not lead to a significant increase in the cytotoxic activity (Figure 5a). After 48 h, the proliferative activity of cells was reduced by 19–47% under the action of NDGA and baicalein, and by their combinations with nAChR blockers, while zileuton had no such effect (Figure 5b). After 72 h, the effects of NDGA and baicalein were more pronounced than those at 48 h; α7 nAChR blockers reduced the antiproliferative effect of baicalein to some extent (Figure 5b). Thus, baicalein and zileuton produced statistically significant time dependent decrease in cell viability; however, these effects were not influenced by nAChR blockers.

To investigate the relation of these effects to the COX activity, we used the nonselective COX inhibitor indomethacin or the selective COX-2 and COX-1 inhibitors NS398 and CS560, respectively. It was shown that the antiproliferative effect of NDGA was preserved when this drug was used together with a nonselective COX inhibitor indomethacin (11% reduction in cell concentration) (Figure 5c). The addition of CTX reduced the antiproliferative effect of NDGA which became weak, in contrast to conditions when the activity of COX (Figure 5b) or activity of LOX (Figure 4b) was not inhibited. Interestingly, the use of a selective COX-2 inhibitor NS398, but not a COX-1 inhibitor CS560, canceled the antiproliferative effect of NDGA (Figure 5c). When the toxins [V11L,V16D]ArIB or CTX were added there too, the proliferative activity of cells was decreased or showed the tendency to decrease (Figure 5c).

In general, these results showed that LOX inhibitors, and their combination with nAChR blockers, reduced the cell viability by 5–19%, however in some cases without statistical significance. Nevertheless, the nonspecific LOX inhibitor NDGA and the 12-LOX inhibitor baicalein exerted statistically significant antiproliferative effects on the C6 glioma cells, and this effect was enhanced with an increase in in the cultivation time from 24 to 72 h. Blockers of α7 nAChR reduced the effect of NDGA after 24 h of incubation only and, to a small extent, the effect of baicalein after 72 h only.

As described above, suppression of proliferation and a decrease in viability were observed at nicotine concentrations of 1 μL/mL (6.1 mM) (Figure S2). The blockade of nAChR with α-conotoxins or CTX at concentrations up to 100 nM did not cancel the effects of nicotine (Figure S3). COX inhibitors did not change the cytotoxic effect of nicotine, while baicalein, NDGA, and its combination with indomethacin slightly reduced it (Figure S4a). As for the antiproliferative effect of nicotine, the COX and LOX inhibitors practically did not change it (Figure S4b). However, as mentioned earlier, the apparent antiproliferative effect of nicotine may be mediated by the detachment of cells from the substrate.

### 2.4. Detection of nAChR Subunits in C6 Glioma Cells by Quantitative Real-Time PCR

**Figure 6:**
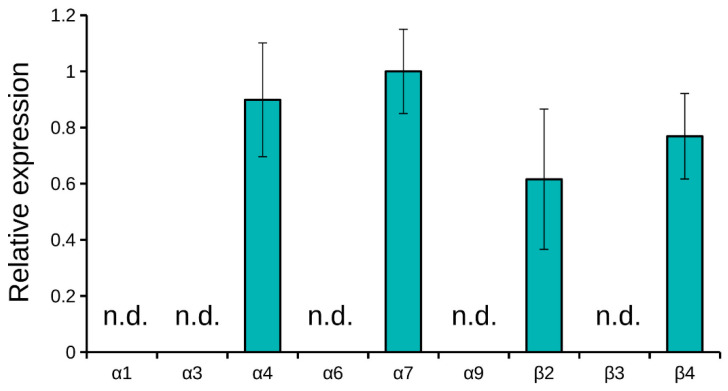
Real-time PCR detection of mRNA for nAChR subunits. Only α4, α7, β2 and β4 subunits were detected. ΔCT values normalized to the α7 ΔCT value are shown. Results are presented as mean ± SE of two independent experiments. Not detected, n.d.

In order to identify the mRNA expression of several nAChR subunits in the C6 glioma cells, quantitative real-time PCR was used. We looked for the subunits which constitute the nAChRs selectively inhibited by the toxins studied (Table 1). We screened the expression of mRNA for of α1, α3, α4, α6, α7, α9, β2, β3 and β4 nAChR subunits. At mRNA level, only four types of subunits were detected via real-time PCR: α4, α7, β2 and β4 (Figure 6). After the incubation with high nicotine concentration, no signs of mRNA for nAChR subunits were detected.

### 2.5. Detection of nAChR in C6 Glioma Cells by Radioligand Analysis

**Figure 7:**
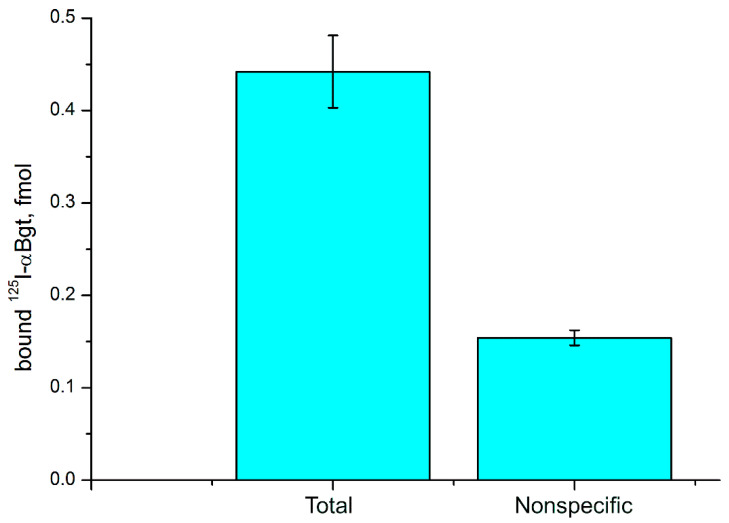
Binding of radioactive ^125^I-labeled α-bungarotoxin (^125^I-αBgt) to glioma C6 cells. Nonspecific ^125^I-αBgt binding was determined in the presence of 200-fold excess of CTX. Results are presented as mean ± SE of two independent experiments (n = 5 in each experiment).

To check if C6 glioma cells contain nAChRs, we applied radioligand analysis. Radioactive α-bungarotoxin, which is selective to neuronal α7 and α9 nAChRs, was used as a ligand. It was found that α-bungarotoxin bound to C6 cells and this binding was inhibited by the excess of CTX (Figure 7). These data suggest the presence in glioma cells of either α7 or α9 nAChR subtype.

## 3. Discussion

The experimental data available at present indicate an opposite role of nAChRs in tumor pathogenesis and the response to nAChR agonists and antagonists may significantly depend on the cell type. Many studies support the view that activation of nAChRs has a protumor effect. Thus, epidemiological studies show that the agonists of these receptors, nicotine and its derivatives contained in tobacco smoke, contribute to the onset and development of tumors of the lung [1], breast [3,63], pancreas [64,65], head and neck [66], accelerating proliferation and suppressing apoptosis. On the contrary, blockade of nAChRs slows tumor growth in experimental models of lung [21,22] and breast [18,20] cancer. The effect of nicotine is realized with the participation of nAChRs containing α7 [1,67], α9 [1,15,68] and α5 [1,2,16] subunits.

However, a number of publications have disclosed the cytotoxic and antiproliferative effects of nicotine: nicotine or its active derivatives induced apoptosis and arrest of the cell cycle in podocytes [23,24], osteoblasts [25], human renal proximal tubular epithelial cells (HRPTEpC) [26] and in human hepatocellular carcinoma cell line [27]. Nicotine reduced the proliferation of human Wharton’s jelly mesenchymal stem cells [69], rat microglia [70] and periodontal ligament-derived stem cells [71]; it caused autophagy in the Leydig cells [72] and pyroptosis of human aortic endothelial cells (HAECs) [73]. Nicotine affects embryonic mouse stem cells in two ways: at low concentrations (0.01 and 0.1 μM) it stimulates, while at high concentrations (1 and 10 μM) it suppresses proliferation without affecting viability [74].

Our results show that when nAChRs are inhibited by α-conotoxins or CTX, proliferation of C6 glioma cells is activated. The most consistent effects were observed for CTX and [V11L,V16D]ArIB, which suggest the inhibition of α7 nAChR. A statistically significant increase in C6 cell proliferation was observed in the presence of α-conotoxin PnIA selective for α3β2 nAChR. A smaller but statistically significant effect was found after 24 h of incubation with RgIA selective for α9α10 nAChR. Acetylcholine at concentrations up to 2 mM produced no effects. However, it should be borne in mind that acetylcholine can be degraded very fast by cell enzymes during incubation. Nicotine is an agonist of the most nAChR subtypes with the exception of α9α10 nAChRs for which it is an antagonist [75]. Nicotine showed no effect at concentrations up to 0.1 µL/mL (0.61 mM). While at 1 µL/mL (6.1 mM), it reduced cell proliferation, the cell exhibited morphologic changes and detached from the substrate. The last effects might be the reason for the apparent decrease in proliferation. This suggestion was confirmed by our experiments on the blockade of nAChR, which did not lead to the cancellation of the antiproliferative effect of nicotine, and its cytotoxicity even increased. It means that the effects of nicotine at high concentrations are not related to the activation of nAChRs and are mediated by other mechanisms. Thus, C6 glioma cells may not be sensitive to nicotine; that is, activation (or blockade) of nAChRs by nicotine may not affect C6 glioma cells. Another possibility may be that the nAChRs in these cells are not sensitive to nicotine. The ionotropic acetylcholine receptors insensitive to nicotine were found in nematodes *Caenorhabditis elegans* [76] and *Haemonchus contortus* [77], and in sea hare *Aplysia californica* [78]. Despite the absence of nicotine effects, our data for α-conotoxins and CTX show that in glioma C6 cells, nAChRs are involved in the pathways for regulation of survival and proliferation. The inhibition of nAChRs resulted not only in the increase of proliferation, but also in a slight decrease in cell viability. This suggests the activation of pathways that are involved in both proliferation and cell death. For example, as it takes place in the case of apoptosis-induced proliferation (so called compensatory proliferation), which is stimulated in tissues during cell death and proceeds with the participation of caspases [79]. It should be noted that activation of nAChRs can result in inhibition of cell proliferation; this effect was shown for human hepatocellular carcinoma (HepG2) cell line [27], human podocytes [23] and rat microglia [70]. Although nicotine was inactive in our case, it was used as an nAChRs activator in the above studies, and nAChR activation produced the negative effect on cell proliferation. It may be possible that nAChR inhibition enhances proliferation. Our study has demonstrated similar effects. It can be assumed that the physiological activity of nAChRs is sufficient to negatively regulate the proliferation of C6 glioma cells, as the blockade of nAChR cancels this regulation and enhances the cell proliferation. Therefore, when only nicotine is added, an additional activation does not affect proliferation. It should be mentioned that in brain neurotransmitter including acetylcholine can exert their effects through so called extrasynaptic communication acting on the receptors located outside synapses. Extrasynaptic receptors can be activated by neurotransmitter that escapes the synapse or diffuses through the extracellular space from distant sites [80]. Therefore, the presence of the nAChRs on glial cells from which glioma originated should not be unexpected. Furthermore, analysis of the Repository for Molecular Brain Neoplasia Data, carried out in [47], showed that the genes of choline transporters, choline acetyltransferase, and vesicular acetylcholine transporters are expressed in human glioblastomas. This suggests that glioblastoma cells can synthesize endogenous acetylcholine [47]. Moreover, it was shown that C6 cells contained significant amounts of acetylcholine [50]. Thus, the endogenous acetylcholine present in these cells may activate their nAChRs.

To detect nAChRs expressed in the C6 glioma cells, we used the eight nAChR subunit gene specific primers for real-time PCR and found that mRNA for only four subunits, i.e., α4, α7, β2 and β4, were expressed at the detectable levels. The presence of mRNA for these subunits might indicate the expression of receptor containing these subunits at the protein level. Earlier, the presence of α4β2 nAChRs was shown in glial cells of adult rat brain [81], of α7 nAChR in cultured rat cortical microglia [82] and of α4, α7, β2, β3 subunits in the primary culture of rat astrocytes [49]. It was shown that primary cultures of rat astrocytes expressed mRNA for nAChR subunits [49], but since mRNA encoding corresponding subunit in the brain was used as control, it is not possible to use these data for quantitative comparison of the mRNA expression level in normal and glioma cells. Nevertheless, comparative data for the expression of nAChR mRNA in normal and tumor cells were reported [47]. In the samples of patients with glioblastomas, increased expression of mRNA for α1, α9 and β1 subunits was detected, while the mRNA expression of many other nAChR subunits was decreased. However, it should be borne in mind that the normal samples included a large population of neurons, which are characterized by increased expression of nAChR. On glioma C6 cells, we observed the effects of α-conotoxin [V11L,V16D]ArIB specific to α7 and of CTX, which among neuronal AChRs acts on α7 and α9 subtypes. The binding to glioma cells of ^125^I-αBgt, which is specific to neuronal AChRs of α7 and α9 subtypes, was found using radioligand assay. The effects of α-conotoxin PnIA selective for α3β2 and α6/α3β2β3 nAChR on the C6 cell viability and proliferation were observed. These data indicate possible involvement of homomeric α7 and heteromeric β2-containing nAChRs in proliferation of glioma. Based on the data of real-time PCR, radioligand assay and specific effects of α-conotoxins, we can conclude that α4, α7, β2 and β4 subunits are present in glioma cells. As α-conotoxin PnIA interact with heteromeric β2-containing nAChRs, its effect may be explained by the action either on α4β2 subtype with which this α-conotoxin interacts very weakly (IC_50_ > 1 µM [83]) or on the supposed heteromeric α7β2 subtype of nAChR. The presence of this nAChR subtype in rodent brain was shown earlier [84,85]. It binds CTX with same affinity as homomeric α7 nAChR [86], but there are no data about its interaction with α-conotoxins. However, α7β2 subtype of nAChR demonstrated a high affinity to dihydro-β-erythroidine, β2 subunit-containing nAChR-selective antagonist [87]. Interestingly, after preliminary incubation of C6 cell with nicotine, no mRNA for nAChR subunits was detected by real-time PCR. The fact may be explained by downregulation of the mRNA expression in the presence of agonist. A similar downregulated expression of mRNAs for the nAChR subunits in the presence of nicotine was observed earlier, for example, in human leukemic T-cell line [88].

Increased expression of COX-2 and LOX [29,33,35,89] is observed during tumor growth, and a number of compounds of the arachidonic acid cascade, including prostaglandin E2 and leukotrienes, support the survival and proliferative activity of tumor cells, promote tumor growth, accelerate tumor angiogenesis and suppress antitumor immunity. Based on this, the possibilities of tumor therapy and prevention of tumor formation using inhibitors of eicosanoid synthesis are currently being actively studied.

Nonsteroidal anti-inflammatory drugs (NSAIDs) are the most common COX inhibitors. Experimental studies and clinical observations have shown that NSAIDs exhibit an antitumor effect by reducing tumor formation, and in some cases slowing the growth of tumors [39,90,91]. Inhibitors of COX-1 [92] and COX-2 [93,94] reduce the viability and proliferative activity of tumor cells, including human gliomas [93]. This may be caused by inhibition of prostaglandin E_2_ synthesis due to a decrease in the catalytic activity of COX or by COX-independent mechanisms [93,94].

LOX inhibitors also have antitumor effects; 5-LOX inhibitors, e.g., zileuton, are the promising NSAIDs with an antitumor effect in various models both in vitro and in vivo [95]. Double inhibition of COX-2 and 5-LOX in animal experiments leads to an even more pronounced effect on different tumor cells [35] and also potentiates the antitumor effect of the antimelanoma vaccine [96]. Baicalein, a 12-LOX inhibitor, suppresses the transformation and proliferation in some cancers [97].

In our experiments, C6 glioma cells were highly sensitive to the nonselective LOX inhibitor NDGA and the selective 12-LOX inhibitor baicalein, with a weak response to the selective 5-LOX inhibitor zileuton. This suggests that 12-LOX plays a significant role in the survival and especially in the proliferative activity of C6 glioma cells. Earlier, we observed similar effects of LOX inhibitors in the Ehrlich’s ascites carcinoma cells [20]. Unexpectedly, our data (Figure 5c) show that the selective COX-2 inhibitor NS-398 reduced the antiproliferative effect of NDGA. It means that COX-2 may well participate in the antiproliferative effect of NDGA.

Another question that we wanted to answer was a possible involvement of nAChRs in the effects of NDGA and baicalein, and, reciprocally, the participation of COX and LOX in the effects of the nAChR agonists and antagonists. It seemed probable that the antiproliferative action of NDGA and baicalein involves α7 nAChRs, since blockers of these receptors reduce the antiproliferative effect of NDGA. It was earlier shown that the activation of nAChR is accompanied by an increase in the expression of COX-2 in human gastric cancer cells [4,5]. It is possible that in C6 glioma cells, the blockade of nAChR downregulates the expression of COX-2, and this is accompanied by a decrease in the antiproliferative effect of NDGA. However, a more detailed study is necessary to bring more clarity to the mechanisms of interaction between nAChR and enzymes of the arachidonic acid cascade.

The pro-proliferative effect of α7 nAChR blockers was manifested upon inhibition either of COX or LOX, but it was decreased upon simultaneous inhibition of both groups of enzymes. This means that to enhance proliferation at the blockade of nAChR, the participation either of COX or LOX is required. It should also be noted that at the blockade of nAChR, an increase in proliferation was observed after 24 and 48 h, but was not detected after 72 h. That is, C6 glioma cells were adapted to blockers, and this may be due to a change in the expression of nAChR, the development of other physiological mechanisms of cell adaptation, or proliferation of cells that were initially present in a genetically heterogeneous population and were more resistant to nAChR blockade.

## 4. Materials and Methods

### 4.1. Materials

Solid-phase peptide synthesis was used to obtain α-conotoxins [V11L,V16D]ArIB [54], MII [98], PnIA [99] and RgIA [100]. α-Cobratoxin was isolated by the procedure described in [53] from Thailand cobra venom obtained from an authorized farm (Vinh Son, Vinh Tuong, Vinh Phuc province, Vietnam). Baicalein, indomethacin, NDGA, NS-398, SC-560, (–)-nicotine and zileuton were acquired from Merck KGaA (Darmstadt, Germany). Rat glioma C6 cells were obtained from the Republican Research and Practical Center for Epidemiology and Microbiology (Minsk, Republic of Belarus).

### 4.2. Assay of the Viability and Proliferation of C6 Glioma Cells

Dulbecco’s modified Eagle’s medium (DMEM, Merck KGaA, Darmstadt, Germany) with the addition of antibiotics (penicillin, streptomycin and amphotericin B; all from Merck KGaA, Darmstadt, Germany) and 10% HyClone™ fetal bovine serum (FBS, Thermo Fisher Scientific, Waltham, MA, USA) was used for the preparation of cell suspensions with a concentration of 0.20 million cells/mL (for analysis after 24 h), 0.10 million cells/mL (for analysis after 48 h) and 0.04 million cells/mL (for analysis after 72 h). During the experiments, into the wells of one of the 96-well culture plates (Costar, Corning Inc, Corning, NY, USA), 200 µL of a cell suspension with a concentration of 0.20 million cells/mL were seeded; into the wells of the second plate, a concentration of 0.10 million cells/mL; and into the wells of the third plate, a concentration of 0.040 million cells/mL. Afterward, COX or LOX inhibitors, nicotine, nAChR blockers or solvents for these drugs were added at a volume of 25 μL each. Saline was used to dissolve nAChR blockers, DMEM with 10% FBS for nicotine, and DMSO for COX and LOX inhibitors. The required volume was adjusted with DMEM containing 10% FBS. Final concentrations of nAChR blockers were 1 and 100 nM. The following final concentrations COX or LOX inhibitors were used: indomethacin—10 μM, SC-560—0.1 μM, NS-398—5 μM, NDGA—30 μM, zileuton and baicalein—50 μM. A similar amount of solvent was added in the control series. In all wells, the final concentration of DMSO was 0.1%. Nicotine was used at final concentrations of 0.001–10 µL/mL. The combined data of several experiments are shown; in each series, n = 4–6. The plates with cells were kept in a CO_2_ incubator at 5% CO_2_ and 37 °C. The cells from the first, second and third plates were collected in flow cytometry tubes, after 24, 48 and 72 h, respectively. Staining with propidium iodide (Sigma) was applied to evaluate cell viability. Flow cytometer BD FACSCanto II with Diva 7.0 software (Becton Dickinson, Franklin Lakes, NJ, USA) was used for the cell sample analyses. Cell proliferative activity was determined by calculating the cell concentration in the samples using FLOW-COUNT™ reference fluorospheres (Beckman Coulter, Inc, Brea, CA, USA).

### 4.3. MTT Assay

Glioma C6 cells (4000 cells/well) were seeded in the 96-well culture plates. The solutions of CTX (at final concentrations of 3 pM–3 µM) or cytotoxin I *Naja oxiana* (final concentration of 1 µM) in the DMEM growth medium were added to the wells and the plate incubated 72 h at 37 °C in a humidified atmosphere with 5% CO_2_. Then, 20 μL of MTT solution at a concentration of 2 mg/mL was added to each well and the plate was further incubated for 2 h. Thereafter, growth medium was aspirated and replaced by 100 μL of dimethyl sulfoxide and cell viability was evaluated by measuring the optical density of the resulting formazone solution using a Hidex Sence plate spectrophotometer (Hidex, Turku, Finland) at wavelengths of 570–700 nm. The data were analyzed using Hidex Sense software (Hidex, Turku, Finland) and OriginPro 7.5 software (OriginLab, Northampton, MA, USA).

### 4.4. Light Microscopy

For microscopy, C6 glioma cells were plated in 35 mm Petri dishes (Corning Inc., Corning, NY, USA), 0.1 million cells in 2 mL of medium. Nicotine was added at final concentrations of 0.001, 0.01, 0.1, 1 and 10 μL/mL, in the control–solvent (DMEM medium with 10% FBS). After 24, 48 and 72 h, microscopy was performed using an NU2E inverted phase contrast microscope (Opton, Oberkochen, Germany) with a Nicon LWD 40 × 0.55 objective (Nikon Corporation, Tokyo, Japan). For photography, an XLI-14A camera from XL Imaging (XL Imaging LLC, Carrollton, TX, USA) with the supplied software was used.

### 4.5. Quantitative Real-Time PCR

Total RNA was isolated using trizol extraction (Evrogen, Moscow, Russia). RNA (1 μg) was reverse-transcribed using MMLV revertase kit (Evrogen, Moscow, Russia) and analyzed in qPCR to identify which nAChR subunit was expressed in the three preparations.

Each amplification reaction (10 μL) contained 2 × Sso Advanced Universal SYBR Green Supermix (5 μL), cDNA template (2 μL), forward and reverse primers (500 nM each) and nuclease-free H_2_O (2.5 μL). Everything was used according to the manufacturer′s protocol (Bio-Rad Laboratories, Hercules, CA, USA). cDNA samples were amplified with the following primers: Chrna1 forward 5′-GCCCGACCTGAGTAACTTC-3′ and reverse 5′-CCACTCCTCAGACGCATTG-3′; Chrna3 forward 5′-GCTGTGAGGAGATCTACCAAGAC-3′ and reverse 5′-TCACCATGGCAACGTACTTCC-3′; Chrna4 forward 5′-CATAGCCGTGTGGACCAACTG-3′ and reverse 5′-ACATTGAGCACGAAGACCGT-3′; Chrna6 forward 5′-CTGGAAGCCTGACATCGTTC-3′ and 5′-GATGGTCTCTGTAATCACCAGC-3′; Chrna7 forward 5′-TGGCTACAAATGTCTTGGACAG-3′ and reverse 5′-TCAGGAGAATGATTCTGGTCCAC-3′; Chrna9 forward 5′-GCTTCTGGAATCAGAGCCG-3′ and 5′-CGTCAGCCTTGTTGTATAGGAC-3′; Chrnb2 forward 5′-GGTGGGCAAGTACCTCATGTTTA-3′ and reverse 5′-CGATCACCATGGCAACGTATT-3′; Chrnb4 forward 5′-CATGGTTCTTAAGTCGCCCAC-3′ and reverse 5′-CACCCTCTAATGCTTCCTGTAG-3′; succinate dehydrogenase (SDHA) forward 5′-ATGGGCGAACCTACTTCAGC-3′ and reverse 5′-TACCTGTGGGGTGGAACTGA-3′. Primers were designed using the FastRCR 6.6 software (PrimerDigital Ltd., Helsinki, Finland) and supplied by Evrogen (Moscow, Russia).

The thermal cycling conditions included 2 min at 95 °C, then 20 s at 95 °C, 20 s at 62 °C and 20 s at 72 °C for 36 cycles, followed melting curve measurement from 65 °C to 95 °C. All reactions were performed in the C1000 Touch Thermal Cycler (Bio-Rad Laboratories, Hercules, CA, USA) in duplicates. The reference gene (housekeeping) was SDHA. The levels of nAChR transcripts were estimated using ΔC_T_ values normalized to the α7 ΔC_T_ value.

To determine the effect of nicotine, the cells were incubated with 100 μM of this substance for 72 h before PCR procedure.

### 4.6. Toxin Binding Assay

Glioma C6 cells (3 × 10^5^ cells) were incubated in 50 μL 20mM Tris-HCl buffer (1 mg/mL of BSA, pH 8.0) containing protease inhibitor cocktail with radioactive ^125^I-labeled α-bungarotoxin (1000 Ci/mmol) at the final concentration of 0.22 nM for 2 h at room temperature. The cells were harvested by filtration through glass GF/C filters (Whatman, Maidstone, UK) pretreated with 0.3% polyethylenimine and washed (3 × 4 mL) with cold 20 mM Tris-HCl buffer, pH 8.0, containing 0.1 mg/mL BSA. The nonspecific binding was determined in the presence of 200-fold excess of CTX. The radioactivity bound was measured with a Wallac 1470 Wizard Gamma Counter (PerkinElmer, Waltham, MA, USA).

### 4.7. Statistical Analysis

Statistical analysis was performed using standard statistical methods from the Statistica 7 (TIBCO Software Inc., Palo Alto, CA, USA) and Excel 2010 (Microsoft, Redmond, WA, USA) software packages. The significance of differences between the series experiments is indicated according to the Student test. Differences between the series were considered significant at *p* < 0.05.

## 5. Conclusions

Gliomas are highly malignant and difficult to treat tumors, which necessitates the study of their growth mechanisms and a search for new methods of treatment. In this work, the effect of separate and combined use of two groups of agents on the survival and proliferation of C6 glioma cells was studied. The first group included nAChR antagonists α-conotoxins and CTX, and in the second group were inhibitors of cyclo- or lipoxygenases. It was shown that nAChR blockers promoted the proliferation of C6 glioma cells. Nonspecific LOX inhibitor NDGA and 12-LOX inhibitor baicalein exerted antiproliferative and cytotoxic effects on tumor cells, while nAChR blockers slightly reduced this effect after 24 h of culture. Quantitative real-time polymerase chain reaction showed that mRNA for α4, α7, β2 and β4 subunits of nAChR were expressed in C6 glioma cells. The results obtained revealed new possibilities for regulating the growth of gliomas using physiologically active compounds of natural origin and their synthetic analogs, and also provided new evidence on the regulatory role of nAChRs and enzymes of the arachidonic acid cascade in the survival and proliferation of nerve system tumors.

## Figures and Tables

**Table 1:** Specificity of toxins studied for neuronal nAChRs.

Toxin	nAChR Subtype	Reference
CTX	α7; α9α10	[52,53]
α-Conotoxin [V11L,V16D]ArIB	α7	[55]
α-Conotoxin MII	α3β2; α6-containing	[56,57]
α-Conotoxin PnIA	α3β2; α6/α3β2β3	[58,59]
α-Conotoxin RgIA	α9α10	[60]

## Data Availability

All data is contained within this article and supplementary materials.

## Supplementary Materials

The following are available online at https://www.mdpi.com/1660-3397/19/2/118/s1, Figure S1: The effect of α-conotoxins MII; PnIA; RgIA; [V11L,V16D]ArIB ([L,D]ArIB) and α-cobratoxin (CTX) at concentration of 100 nM on the viability (**a**) and on the proliferative activity (**b**) of C6 glioma cells (n = 6). The cell viability and concentration were determined by flow cytometry. * *p* < 0.05 according to Student’s *t*-test when compared to control. Figure S2: Effect of nicotine (Nic) on the viability (**a**) and proliferation (**b**) of C6 glioma cells (n = 6). The cell viability and concentration were determined by flow cytometry. * *p* < 0.05 according to Student’s *t*-test when compared to control. Figure S3: Effect of nicotine (Nic) at a concentration of 1 μL/mL and α-conotoxins MII; PnIA; RgIA; [V11L,V16D]ArIB ([L,D]ArIB) and α-cobratoxin (CTX) at a concentration of 100 nM on the viability (**a**) and proliferation (**b**) of glioma C6 cells (n = 6). The cell viability and concentration were determined by flow cytometry. * *p* < 0.05 according to Student’s *t*-test when compared to control, ** *p* < 0.05 according to Student’s *t*-test when compared to “Nic”. Figure S4: Effect of COX inhibitors (IM, indomethacin, 10 µМ; NS-560, 5 µM; SC-398, 0.1 µM) or LOX inhibitors (NDGA, nordihydroguaiaretic acid, 30 µM; Bai, baicalein, 50 µM; Zil, zileuton, 50 µM) in combination with nicotine (Nic, 1 μL/mL) on viability (**a**) and proliferative activity of cells (**b**) of glioma C6 cells (n = 12) The cell viability and concentration were determined by flow cytometry. * *p* < 0.05 according to Student’s *t*-test when compared to control, ** *p* < 0.05 according to Student’s *t*-test when compared to “Nic”.
