# Supplementary material for: α-Conotoxins and α-Cobratoxin Promote, while Lipoxygenase and Cyclooxygenase Inhibitors Suppress the Proliferation of Glioma C6 Cells
Source: Mar Drugs. 2021 Feb 21;19(2):118. doi: 10.3390/md19020118 (PMC7956437; doi:10.3390/md19020118)
Supplement: Supplementary file 1 [file marinedrugs-19-00118-s001.pdf]

Supplementary materials

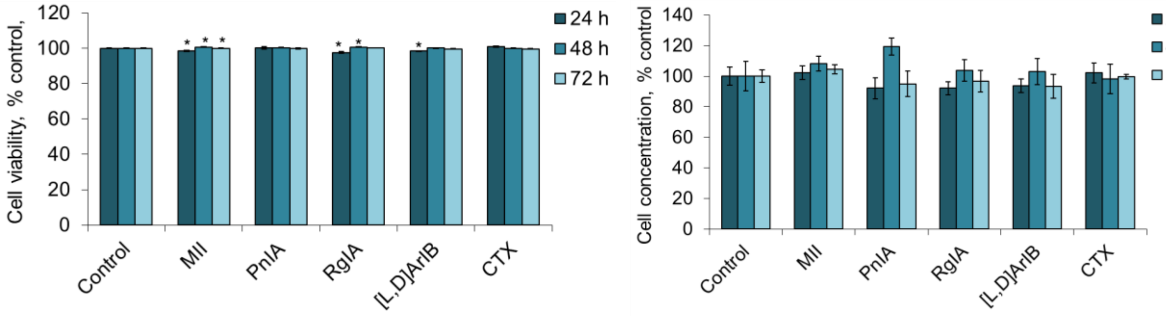

(a) (b)

Figure S1. The effect of α-conotoxins MII; PnIA; RgIA; [V11L,V16D]ArIB ([L,D]ArIB) and α-cobratoxin (CTX) at concentration of 100 nM on the viability (a) and proliferation (b) of C6 glioma cells (n = 6). The cell viability and concentration were determined by flow cytometry. * p <0.05 according to Student's t test when compared to control.

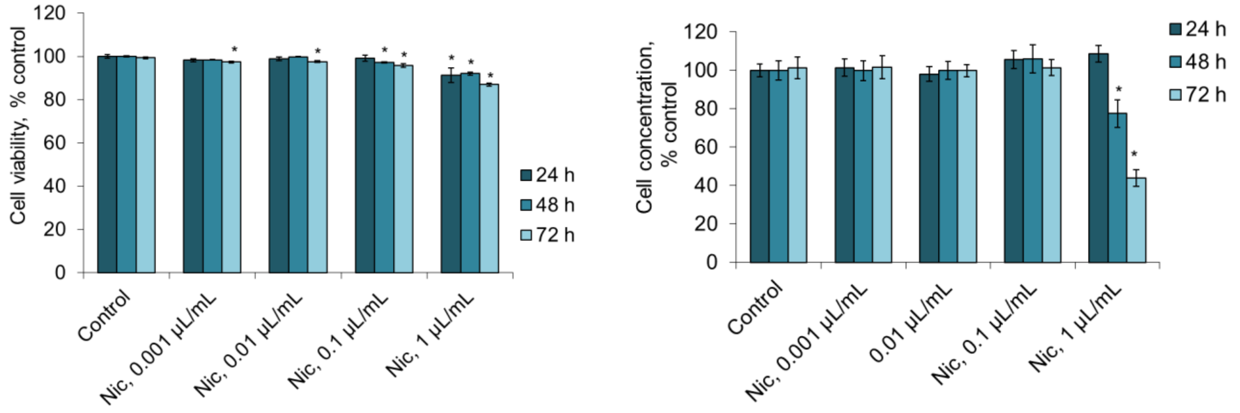

(a) (b)

Figure S2. Effect of nicotine (Nic) on the viability (a) and proliferation (b) of C6 glioma cells (n = 6). The cell viability and concentration were determined by flow cytometry. * p <0.05 according to Student's test when compared to control.

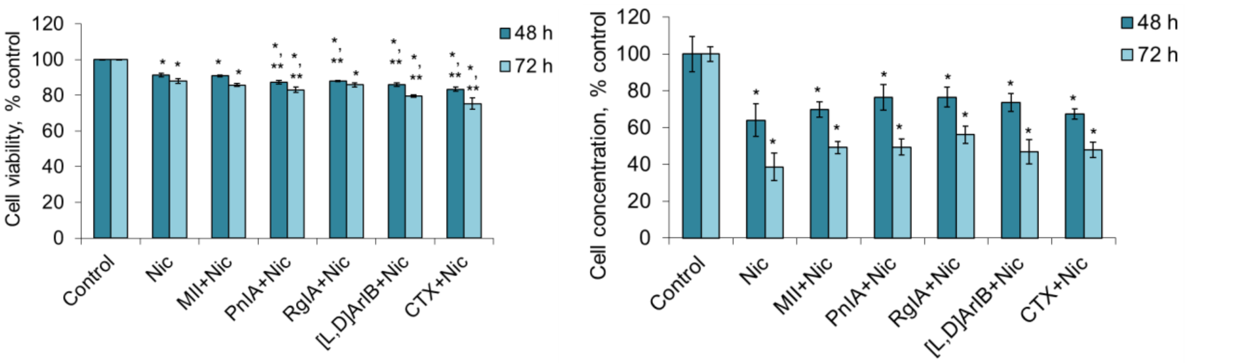

(a) (b)

Figure S3. Effect of nicotine (Nic) at a concentration of 1 µL/mL and α-conotoxins MII; PnIA; RgIA; [V11L,V16D]ArIB ([L,D]ArIB) and α-cobratoxin (CTX) at a concentration of 100 nM on the viability (a) and proliferation (b) of glioma C6 cells (n = 6). The cell viability and concentration were determined by flow cytometry. * p <0.05 according to Student's test when compared to control, ** *p* <0.05 according to Student's test when compared to "Nic".

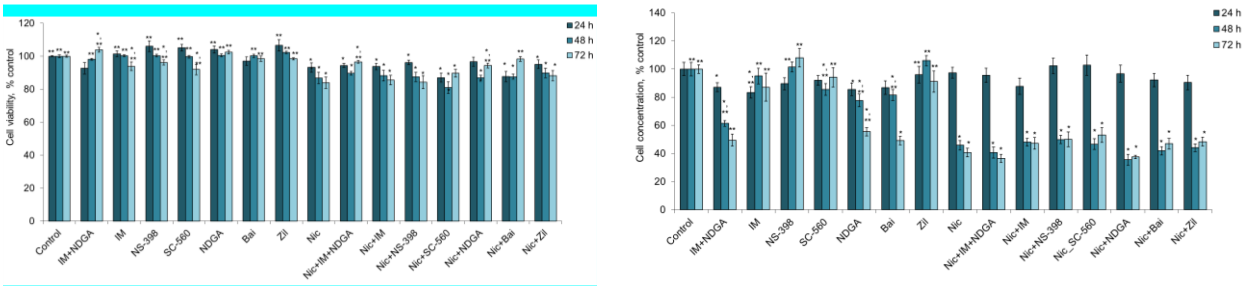

(a) (b)

Figure S4. Effect of COX inhibitors (IM, indomethacin, 10 µM; NS-560, 5 µM; SC-398, 0,1 µM) or LOX inhibitors (NDGA, nordihydroguaiaretic acid, 30 µM; Bai, baicalein, 50 µM; Zil, zileuton, 50 µM) in combination with nicotine (Nic, 1 µL/mL) on viability (a) and proliferation (b) of glioma C6 cells (n = 12) The cell viability and concentration were determined by flow cytometry. *p* <0.05 according to Student's test when compared to control, ** *p* <0.05 according to Student's test when compared to "Nic".
